# High risk of postoperative complications in dialysis patients undergoing total hip arthroplasty: a database study of Japanese nationwide medical claims

**DOI:** 10.1038/s41598-025-02829-8

**Published:** 2025-07-07

**Authors:** Hidetatsu Tanaka, Kunio Tarasawa, Yu Mori, Kazuyoshi Baba, Hideki Fukuchi, Kiyohide Fushimi, Kenji Fujimori, Toshimi Aizawa

**Affiliations:** 1 Department of Orthopaedic Surgery, Tohoku University Graduate School of Medicine, 1-1 Seiryo- machi, Aoba-Ku, Sendai, 980-8574 Miyagi Japan; 2 Department of Health Administration and Policy, Tohoku University Graduate School of Medicine, 2-1 Seiryo-machi, Aoba-Ku, Sendai, 980-8574 Miyagi Japan; 3 Department of Health Policy and Informatics, Institute of Science Tokyo, 1-5-45 Yushima, Bunkyo-Ku, Tokyo, 113-8519 Japan

**Keywords:** Dialysis patients, Total hip arthroplasty, Postoperative complications, Dislocation, Nationwide database, Diagnosis procedure combination (DPC), Medical research, Risk factors

## Abstract

Dialysis patients who develop degenerative hip disease or femoral neck fractures may require total hip arthroplasty, and their comorbidities predispose them to complications. This study aimed to evaluate whether dialysis was associated with early postoperative complications using a large database of Japanese. In this cohort study, using the Japanese National Administrative Diagnosis Procedure Combination database on THA for patients on hemodialysis or not from December 2011 to March 2023, we assessed the surgical-related complications, medical complications, and mortality during hospitalization after propensity score matching by age, sex, BMI, and comorbidities. A total of 2,111 pairs of patients on hemodialysis and non-dialysis were included. In THA for patients on hemodialysis, the significant odds ratios for various complications were as follows: dislocation (2.616, 95% CI: 1.282 to 5.338, *p* < 0.01), reoperation (2.104, 95% CI: 1.222 to 3.623, *p* < 0.01), deep vein thrombosis (0.407, 95% CI: 0.286 to 0.579, *p* < 0.01), cerebrovascular events (4.426, 95% CI: 1.495 to 13.10, *p* < 0.01). These findings help identify postoperative THA risks for patients on dialysis, suggesting that more attention should be paid to preoperative planning and postoperative care.

## Introduction

The number of patients on dialysis in Japan continues to increase every year, reaching 347,671 at the end of 2020 and giving a prevalence rate of 2,754 patients per million population. There were 40,744 incident dialysis patients during 2020^1^. Chronic renal failure patients often have the associated high incidence of joint arthropathy, osteoporosis, and pathologic fractures due to dialysis-related amyloidosis or renal osteodystrophy^2–7^. In the dialysis patients, amyloid deposits in joints and tendon sheaths cause aggressive osteolytic lesions and neurological disorders^8^, and increases the risk of fractures^9^. Dialysis patients have renal osteodystrophy with musculoskeletal dysfunction, increased soft tissue laxity, and decreased muscle tone^10^. Previously, patients on hemodialysis had significant risk factors for poor outcomes and perioperative complications after Total Hip Arthroplasty (THA) for osteoarthritis and osteonecrosis of the femoral head^11–15^. Surgeons are cautious about performing joint replacement surgeries in patients undergoing dialysis due to the high risk of intraoperative complications.

Among the orthopedic surgeries, THA has the highest rates of successful post-surgical outcomes and is the most promising surgical procedure for hip degenerative diseases^16^. Although THA is an effective treatment for dialysis patients, it has been reported that the risk of mortality^11,17–22^, re-admission^11^, deep or superficial infection^10,23^, postoperative dislocation^10,18^, and prolonged length of hospital stay^11,18,24^. On the other hand, it has been reported that dialysis patients are not at risk of infection and dislocation for patients on dialysis with osteonecrosis of the femoral head^11^. Dialysis was not a risk of medical complications such as postoperative pneumonia, embolism, or cardiac events^18^. Patients on dialysis tend to have many comorbidities. However, few large-scale studies have compared the complexity of post-THA with those of non-dialysis patients. Early postoperative complications may occur due to bone fragility and muscle weakness induced by dialysis-associated amyloidosis and renal osteodystrophy.

Therefore, this study evaluated whether THA for dialysis patients with hip degenerative diseases and femoral neck fracture was associated with early postoperative complications compared to THA for non-dialysis patients using the Diagnosis Procedure Combination (DPC) database for Japanese. Specifically, we analyzed (1) hip and surgical-related complications, (2) medical complications, and (3) mortality during hospitalization with the large database, adjusting the balance of background information.

## Materials and methods

This study was conducted using data from the Japanese Diagnosis Procedure Combination (DPC) database with the ethical standards of the Declaration of Helsinki and approved by the Institute of Science Tokyo (approval No. M2000-788) and Tohoku University Graduate School of Medicine (approval No. 2021-1-1082).

### Study design

The DPC data in this study included around 1,100 hospitals, covering around 70% of all hospitalization episodes annually in Japan, reflected in the country’s clinical practices. The anonymized data contains the hospital identification number, patient age, gender, diagnosis (coded according to the International Classification of Diseases, Tenth Revision codes (ICD-10), hospital admission and discharge dates, discharge status, drugs, and procedures, including surgery and used drugs. In addition, admission diagnoses, pre-existing comorbidities at admission, and post-admission complications during hospitalization are separately recorded. The Japanese National Administrative DPC Reimbursement System retrospectively reviewed the database. The inclusion criteria were set to (1) THA: receipt code for surgical procedure of 150050410, (2) the disease that required the most medical resources of osteoarthritis of the hip with ICD code of M160-169, (3) osteonecrosis of femoral head with ICD code of M8705, M8715, M8725, M8735, M8785, and M8795, (4) femoral neck fracture with ICD code of S7200 from December 2011 to March 2023 on the DPC database. A total of 332.386 patients were included in the study. There were 2,143 patients (0.64%) on long-term hemodialysis (new dialysis patients not included).

**Fig. 1:**
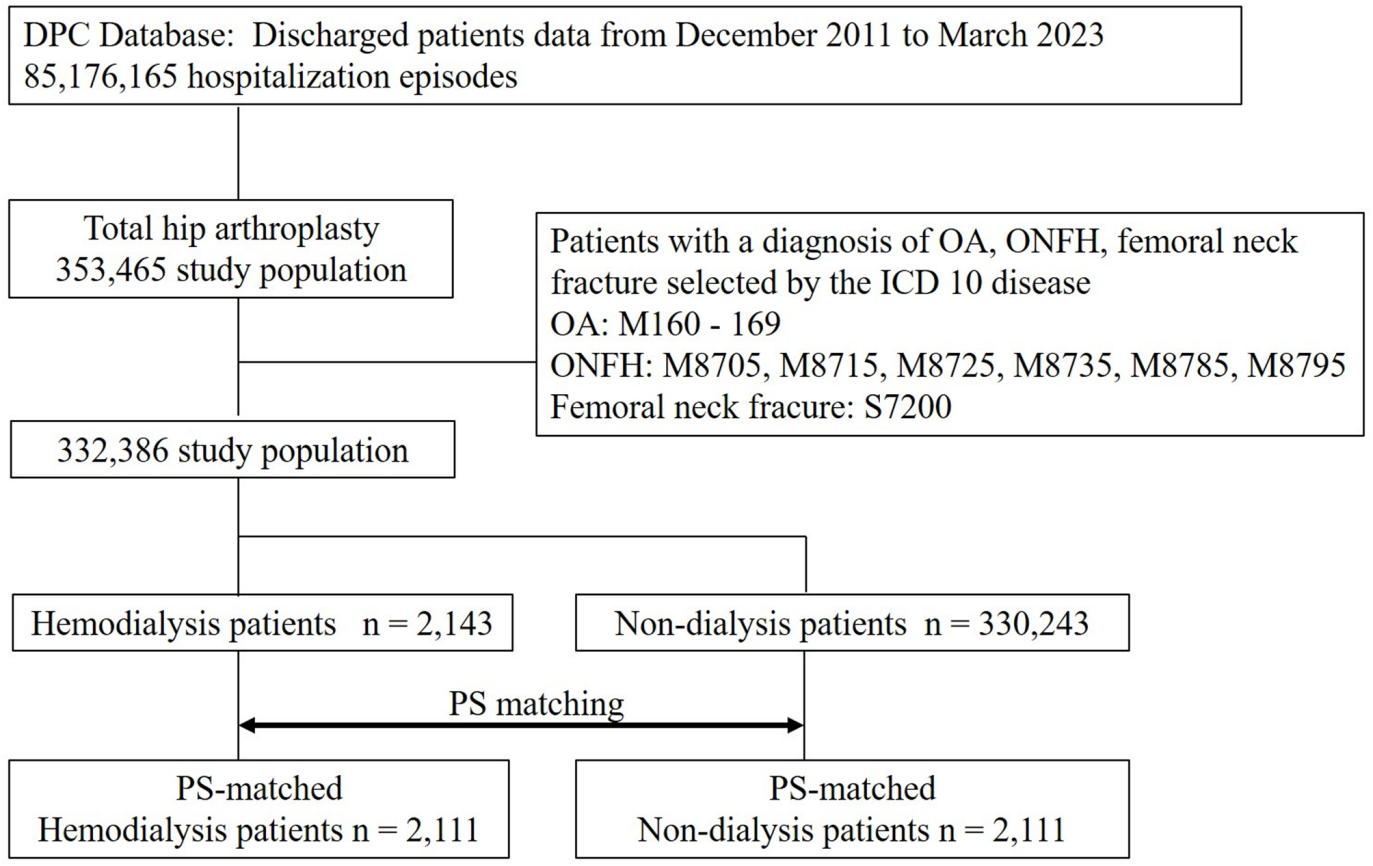
Study flow chart.

Baseline demographic data, including age, gender, BMI, surgical side, diagnosis for THA, comorbidities, length of stay, transfusion rate of day 0 (same day as surgery), day 1 and daiy2, osteoporosis treatment, postoperative anticoagulation, usage of bone cement, and computer-assisted surgery including navigation system including robotic assist, navigation, and portable navigation was shown Table 1. A flow chart of the present study is shown in Fig. 1.

### Data selection

One-to-one Propensity Score (PS) matching was performed between the patients on hemodialysis and non-dialysis. Covariates used for confounding settings included age, gender, BMI, one or both surgical sites, and the most medical resources for THA, cerebrovascular disease, ischemic heart disease, chronic pulmonary disease, hypertension, diabetes, hyperlipidemia, and liver cirrhosis. C-statistics were used to assess the discriminative power of the models. PS estimates were used to perform nearest-neighbor matching without replacement, with the PS estimates being used as the calipers; the caliper was set to 0.2 times the standard deviation of the PS estimate. This resulted in matched pairs and the establishment of PS-matched hemodialysis and non-dialysis groups.

### Outcomes

Assessed outcomes included postoperative dislocation, infection, periprosthetic fracture, and reoperation during hospitalization as surgical complications. Furthermore, the development of hospital-acquired pneumonia, deep vein thrombosis (DVT), pulmonary embolism (PE), cardiac event, cerebrovascular event, sepsis, and mortality during hospitalization as medical complications have been investigated. These complications were compared between hemodialysis and non-dialysis.

### Statistical analysis

All data are expressed as mean ± standard deviation. Significant differences between the two groups were examined using the χ2 test and Student’s t-test for each parameter. The Shapiro-Wilk test was employed to assess the normality of variable distributions. Univariate logistic regression analysis examined the relationship between two joint diseases and the incidence of dislocation, infection, periprosthetic fracture, reoperation, pneumonia, DVT, PE, cardiac events, cerebrovascular events, sepsis, and in-hospital mortality. Following the initial variate analyses, a multivariate logistic regression analysis was performed. All statistical tests were two-tailed; p-values < 0.01 were considered significant. All analyses were performed using JMP version 17.2 (SAS, Cary, NC, USA).

## Results

After one-to-one PS matching, 2,111 patients were on hemodialysis and non-dialysis. Baseline demographic data is shown in Table 2. The C statistic was 0.7213. Standardized mean differences (SMD) were < 0.1 for all covariates used for confounding adjustment. In patients on hemodialysis, the average length of hospital stay was significantly longer. The rate of blood transfusion use on DAY 0 was significantly higher in non-dialysis patients, but there was no difference between DAY 1 and DAY 2 after PS matching. The osteoporosis treatment rate was higher in hemodialysis patients. For non-dialysis patients, antiplatelet agent medication rates were significantly higher than antiplatelet agent medication rates. The bone cement was significantly common in dialysis patients. Computer-assisted implantation has been used significantly more frequently in non-dialysis patients.

The associations between hemodialysis and the development of surgical complications are shown in Table 3. For patients on hemodialysis, the risk of dislocation, infection, periprosthetic fracture, and reoperation during hospitalization increased to 2.616 (95% Confidence Interval [CI]: 1.282 to 5.338), 1.484 (95% CI: 0.851 to 2.586), 1.223 (95% CI: 0.459 to 3.261), and 2.104 (95% CI: 1.222 to 3.623), respectively. Significant associations were found in the dislocation and reoperation rates during hospitalization.

The results of the multivariate logistic analysis of the association between dislocation and age, gender, BMI, hemodialysis, and computer-assisted surgery for each variable are presented in Table 4. Hemodialysis was associated with a significantly increased risk of dislocation during hospitalization, with an odds ratio of 4.488 (95% CI: 2.448–8.228). The apparent risk of dislocation was not associated with age, gender, BMI, and computer-assisted surgery.

The outcomes of the multivariate logistic analysis for assessing risk factors for reoperation are shown in Table 5. Hemodialysis and diabetes were significantly elevated factor of reoperation during hospitalization, with a ratio of 3.394 (95% CI: 2.153 to 5.351) and 2.323 (95% CI: 1.512 to 3.570). The apparent risk of reoperation was not associated with age, gender, BMI, and computer-assisted surgery.

The associations between hemodialysis and the development of medical complications are described in Table 6. For THA for patients on hemodialysis, the risk of DVT was significantly low with odds of 0.407 (95% CI: 0.286 to 0.579), and the cerebrovascular event was significantly risk with odds of 4.426 (95% CI: 1.495 to 13.10).

## Discussion

The present large cohort study utilized DPC data from December 2011 to March 2023 to investigate early postoperative complications and death rates for THA patients for matched hemodialysis and non-dialysis patients. Our findings showed THA for hemodialysis patients resulted in 2.616-fold significant increased risk of postoperative dislocation, 2.104-fold increased risk of reoperation, and 4.426-fold increased risk of cerebrovascular event during hospitalization compared to THA for non-dialysis patients, even when specific risk factors for disease are taken into account. This study is one of the few to analyze the early surgical and medical complications using large national databases. Several studies have been conducted in Japan using large-scale data, and our previous research on proximal femoral fractures using DPC data, including this study, has been helpful in understanding the overall medical care image^25–27^.

Postoperative dislocation rates were higher in hemodialysis patients than in non-dialysis patients in this study. The prosthetic hip dislocation is a serious problem that is well known, with the most common indication for revision of total hip arthroplasty^28^. Similar to the results of this study, postoperative dislocation rates were increased in dialysis patients within 2 years compared to non-dialysis patients^10,18,29^. The reason for the increase in the dislocation incidence in dialysis patients was reported to be multifactorial and in all likelihood a manifestation of the underlying renal osteodystrophy with impaired muscular skeletal function, increased soft tissue laxity, and decreased muscular tone 10. In our opinion, dialysis patients have a base of soft tissue and muscle fragility compared to non-dialysis patients, and the surgical intervention likely increased fragility, leading to dislocation. Whereas post-THA dislocation is reported to be multifactorial^30–32^, a minimally invasive approach with a low dislocation rate^33–35^, computed tomography-based navigation system^36,37^ can be an efficient solution for dialysis patients. In particular, applying computer-assisted surgery to patients on dialysis would be an effective tool. In our study, reoperation rates were shown to be higher in patients on dialysis compared to non-dialysis. Since there was no difference in the incidence of infection and fracture, it indicated a difference in the rate of dislocation.

A number of studies have reported the incidence and risk factors of postoperative infections after joint arthroplasty using a large data vase clinical registry^10,23^, suggesting an increased infection risk may be persistent after surgery^10^. A prior retrospective study involving 16 patients on hemodialysis who underwent THA for osteonecrosis of the femoral head reported a 19% rate of deep infection in the hemodialysis group with an average follow-up of 54 months^38^. Hoggard et al. demonstrated hemodialysis was not an independent risk factor for infection in the osteonecrosis of the femoral head population^11^. A study evaluating patients on dialysis who underwent THA for all indications from 2000 to 2009 using the NIS database found dialysis to be a risk factor for deep wound infection, with a 1.16% incidence of deep wound infection in the dialysis group^22^. Our present results revealed that hemodialysis was not the risk factor for infection during hospitalization compared to a matched group with a total infection rate of 1.27%. As the cohort was matched for comorbidities, the non-dialysis group may be cases with susceptibility to infection, and thus, there may not be any difference.

In the study, postoperative peri-implant femoral fracture rate has no differences in patients on hemodialysis and non-dialysis. The standardized incidence ratio of hip fracture in dialysis patients compared with the general population was reported to be 6.2 (95% CI 5.7–6.8) in male patients and 4.9 (95% CI 4.6–5.3) in female patients^39^. Patients with chronic renal failure often have an associated high incidence of joint arthropathy, osteoporosis, and pathologic fractures due to dialysis-related amyloidosis or renal osteodystrophy^2–7^. Amyloidosis and associated bone disease may lead to physical weakness of the hip joint, increasing the risk of frequent falls after long-term dialysis and promoting the risk of hip fracture^40^. The incidence of postoperative periprosthetic fractures is significantly lower in cemented fixations compared to cementless fixations from randomized controlled trials and meta-analysis^41,42^. Although poor bone quality is expected, the reason for the lack of difference in fracture rate in this study may be that dialysis patients had a higher rate of osteoporosis treatment and used more bone cement.

Various medical complications are associated with dialysis, such as cardiovascular and transfusion^18,22^. Furthermore, dialysis is a significant risk factor for mortality after THA^11,17–22^, with a rate of mortality reported to be 0 to 14%^14,15,43^. The cerebrovascular event during hospitalization tended to have a higher complication rate compared to non-dialysis after THA. In patients on hemodialysis, cerebrovascular disease is one of the causes of mortality. The lower risk of DVT in dialysis patients may be related to the type of dialysis. Anticoagulants such as heparin or low molecular weight heparin are generally used during dialysis. The use of heparin reported to reduce the risk of DVT^44^, the use of anticoagulants with dialysis may have reduced the risk of DVT. Nevertheless, in our study, it might be because the matched non-dialysis group included patients with more complications. A few studies reported that THA for patients on dialysis was a not risk factor for DVT^10^, PE^10,18^, pneumonia^18^, cardiac event^18^, and mortality^45^. Ponnusamy et al. recommended that THA in dialysis-dependent patients carry a high risk of complications and hospital mortality and should be postponed until after kidney transplantation if possible^22^. Our matched cohort study showed that although there was a high risk of medical complications, there was no difference in the hospital mortality rate. THA may be recommended for hemodialysis patients if measures are taken to prevent postoperative dislocation.

There are several limitations to the present study. First, the study population included patients who performed THA only in hospitals reporting to the DPC data system. This did not include patients admitted to non-DPC reporting beds, which account for 30% of all general hospital beds. Secondly, the DPC database lacks information about approaches to the hip, previous hip surgeries, implants, X-ray data including severity of the diseases and implant positions, blood test results, duration of dialysis, and underlying disease of dialysis. Although our study analyzed the dislocation rate after THA between the osteonecrosis of the femoral head and osteoarthritis of the hip patients, dislocation rates have been reported to vary depending on the approach and implant positioning. Third, the DPC database is only provided during hospitalization, limiting the postoperative analysis period and the possibility of complications occurring after this period. Nonetheless, this study is one of the largest sample sizes of studies on complications after THA in dialysis patients. This study also provided an overall comparison of dialysis patients and a description of disease-related risk factors that have not been covered in many other studies. Fourth, there are multiple methods of computer-assisted surgery, such as the use of navigation, the use of robots, and so on. These databases investigate whether computer-assisted surgery has been applied or not, but the type of computer-assisted surgery cannot be identified. Fifth, although this study was conducted using large-scale data from Japan, it is unclear whether the results can be generalized to non-Japanese populations. Multinational studies are needed to determine if the results can be generalized to populations other than Japanese.

## Conclusion

The study of nationwide medical claim databases has compared patients on hemodialysis and non-dialysis who undergo THA. We found that postoperative dislocation, reoperation, and cerebrovascular events were significantly higher in patients on hemodialysis. These findings help identify postoperative THA risks for patients on HD and suggest that more attention should be paid to preoperative planning and postoperative care.

**Table 1:** Baseline demographic data.

	Dialysis	Non-dialysis	*p* value
n	2143	330,243	
Age	66.5 ± 10.7	67.4 ± 11.1	< 0.01※
Gender (%)			
Men	837 (39.1)	60,048 (18.2)	< 0.01
Women	1306 (60.9)	270,195 (81.8)	
BMI	22.1 ± 4.3	24.0 ± 5.1	< 0.01※
Surgical side Unilateral (%)	2123 (99.1)	321,000 (97.2)	< 0.01
Surgical side Bilateral (%)	20 (0.9)	9243 (2.8)	
Diagnosis for THA (%)			
Osteoarthritis	1362 (63.5)	289,238 (87.6)	< 0.01
Osteonecrosis	452 (21.1)	28,477 (8.6)	
Femoral neck fracture	329 (15.4)	12,528 (3.8)	
Comorbidities (%)			
Cerebrovascular disease	74 (3.5)	5584 (1.7)	< 0.01
Ischemic heart disease	221 (10.3)	9301 (2.8)	< 0.01
Chronic lung disease	11 (0.5)	1770 (0.5)	1
Hypertension	506 (23.6)	61,648 (18.7)	< 0.01
Diabetes	426 (19.9)	37,015 (12.2)	< 0.01
Hyperlipidemia	102 (4.8)	25,953 (7.8)	< 0.01
Liver cirrhosis	85 (4.0%)	6205 (1.9)	< 0.01
Length of hospital stay	34.3 ± 27.9	26.6 ± 15.9	< 0.01※
Transfusion DAY 0 (%)	659 (30.8)	166,764 (50.5)	< 0.01
Transfusion DAY 1 (%)	394 (18.4)	73,590 (22.3)	< 0.01
Transfusion DAY 2 (%)	110 (5.1)	11,236 (3.4)	< 0.01
Medications (%)			
Osteoporosis treatment	1345 (62.8)	45,745 (13.9)	< 0.01
Postoperative oral anticoagulant agent	424 (19.8)	247,028 (74.8)	< 0.01
Postoperative oral antiplatelet agent	762 (35.6)	24,763 (7.5)	< 0.01
Use of bone cement (%)	519 (24.2)	58,327 (17.7)	< 0.01
Computer-assisted surgery (%)	504 (23.5)	93,026 (28.2)	< 0.01

Age, BMI, and Length of hospital stay are shown as mean ± standard deviation.

P-values of < 0.05 are considered significant by the Student-t test andχ2 test.

※ Student-t test.

**Table 2:** Characteristics of patients after propensity score matching.

	Hemodialysis	Non-dialysis	SMD
n	2111	2111	
Age	66.4 ± 10.7	66.6 ± 11.8	0.020
Gender (%)			
Men	825 (39.1)	873 (41.4)	0.046
Women	1286 (60.9)	1238 (58.7)	
BMI	22.2 ± 4.3	22.2 ± 4.3	0.067
Surgical side (%)			
Unilateral	2091 (99.1)	2080 (98.5)	0.048
Bilateral	20 (1.0)	31 (1.5)	
Diagnosis for THA (%)			
Osteoarthritis	1350 (64.0)	1535 (64.1)	0.003
Osteonecrosis	446 (21.1)	445 (21.1)	
Femoral neck fracture	315 (14.9)	313 (14.8)	
Comorbidities (%)			
Cerebrovascular disease	73 (3.5)	79 (3.7)	0.015
Ischemic heart disease	217 (10.3)	203 (9.6)	0.022
Chronic lung disease	10 (0.5)	11 (0.5)	0.007
Hypertension	501 (23.7)	511 (24.2)	0.006
Diabetes	421 (19.9)	425 (20.1)	0.005
Hyperlipidemia	100 (4.7)	87 (4.1)	0.030
Liver cirrhosis	83 (3.9%)	76 (3.6)	0.034

			*p*-value
Length of hospital stay	34.3 ± 27.9	27.8 ± 17.0	< 0.01※
Transfusion (%)			
DAY 0	648 (30.7)	995 (47.1)	< 0.01
DAY 1	392 (18.6)	437 (20.7)	0.0882
DAY 2	107 (5.1)	81 (3.8)	0.0619
Medications (%)			
Osteoporosis treatment	1327 (62.9)	302 (14.3)	< 0.01
Postoperative oral anticoagulant agent	417 (19.8)	1529 (72.4)	< 0.01
Postoperative oral antiplatelet agent	752 (35.6)	242 (11.5)	< 0.01
Use of bone cement (%)	510 (24.2)	381 (18.1)	< 0.01
Computer-assisted surgery (%)	493 (23.4)	582 (27.6)	< 0.01

Age, BMI, and Length of hospital stay are shown as mean ± standard deviation.

P-values of < 0.01 are considered significant by the Student-t test andχ2 test.

※ Student-t test.

**Table 3:** Association between Hemodialysis and surgical complications.

Complications	Total (*n*)	Univariate analysis		Multivariable analysis		
		Odds Ratio (95% CI)	*p*-value	Odds Ratio (95% CI)	χ2 statics	*p*-value
Dislocation	70	4.479 (2.444–8.205)	< 0.01	2.616 (1.282–5.338)	6.979	< 0.01
Infection	54	1.581 (0.911–2.741)	0.131	1.484 (0.851–2.586)	1.936	0.164
Periprosthetic fracture	18	1.574 (0.609–4.069)	0.480	1.223 (0.459–3.261)	0.162	0.688
Reoperation	106	3.329 (2.117–5.235)	< 0.01	2.104 (1.222–3.623)	7.206	< 0.01

P-values of < 0.01 are considered significant by the χ2 test.

CI means confidence interval.

**Table 4:** Multivariate logistic analysis of risk factors for dislocation.

Variable	Odds Ratio (95% CI)	χ2 statics	*p*-value
Age	1.017 (0.995–1.040)	2.169	0.141
Gender (Men)	1.395 (0.863–2.258)	1.821	0.177
BMI	0.960 (0.906–1.017)	1.938	0.164
Hemodialysis	4.488 (2.448–8.228)	30.31	< 0.01
Computer-assisted surgery	0.770 (0.426–1.394)	0.780	0.377

P-values of < 0.01 are considered significant by the χ2 test.

CI means confidence interval.

**Table 5:** Multivariate logistic analysis of risk factors for reoperation.

Variable	Odds Ratio (95% CI)	χ2 statics	*p*-value
Age	1.003 (0.985–1.021)	0.083	0.774
Gender (Men)	1.179 (0.788–1.763)	0.636	0.425
BMI	0.943 (0.900–0.988)	5.962	0.015
Hemodialysis	3.394 (2.153–5.351)	32.55	< 0.01
Computer-assisted surgery	0.875 (0.545–1.405)	0.314	0.575
Cerebrovascular disease	2.763 (1.323–5.772)	5.916	0.015
Ischemic heart disease	0.918 (0.467–1.804)	0.067	0.801
Chronic lung disease	4.404 (0.959–20.21)	2.602	0.107
Hypertension	0.578 (0.343–0.975)	4.653	0.031
Diabetes	2.323 (1.512–3.570)	13.520	< 0.01
Hyperlipidemia	0.622 (0.191–2.020)	0.716	0.397
Liver cirrhosis	0.892 (0.320–2.490)	0.049	0.825

P-values of < 0.01 are considered significant by the χ2 test;.

CI means confidence interval.

**Table 6:** Association between dialysis and medical complications.

Complications	Total (*n*)	Univariate analysis		Multivariable analysis		
		Odds Ratio (95% CI)	*p*-value	Odds Ratio (95% CI)	χ2 statics	*p*-value
Hospital-acquired pneumonia	12	5.019 (1.098–22.93)	0.038	4.713 (1.029–21.59)	5.315	0.046
DVT	154	0.400 (0.281–0.569)	< 0.01	0.407 (0.286–0.579)	27.23	< 0.01
PE	5	0.250 (0.028–2.235)	0.375	0.283 (0.031–2.579)	1.533	0.263
Cardiac event	3	2.001 (0.181–22.08)	1	1.967 (0.178–21.71)	0.323	0.581
Cerebrovascular event	21	4.530 (1.531–13.41)	< 0.01	4.426 (1.495–13.10)	9.357	< 0.01
Sepsis	46	1.888 (1.026–3.473)	0.053	1.856 (1.005–3.429)	4.084	0.048
Mortality during hospitalization	30	0.841 (0.431–1.640)	0.735	1.222 (0.624–2.392)	0.344	0.559

P-values of < 0.01 are considered significant by the χ2 test.

DVT means deep vein thrombosis; PE means pulmonary embolism; CI means confidence interval.

## Data Availability

The datasets generated and/or analyzed during this study are not publicly available due to their use in other projects but are available from the corresponding author on reasonable request.
